# Comparative Efficacy of Continuous Positive Airway Pressure Versus High-Flow Nasal Cannula Therapy in Children With Acute Bronchiolitis: A Systematic Review and Meta-Analysis

**DOI:** 10.7759/cureus.81826

**Published:** 2025-04-07

**Authors:** Kaushikkumar S Barot, Amulya Adusumilli, Tasleem Fathema, Saswat Kumar Jha, Ilaf M Hamid, Sandipkumar S Chaudhari, Calvin R Wei, Adil Amin

**Affiliations:** 1 Pediatrics, Shantabaa Medical College & General Hospital Amreli, Amreli, IND; 2 Internal Medicine, Mahadevappa Rampure Medical College, Kalaburagi, IND; 3 Medicine, Dr. VRK Women’s Medical College, Aziznagar, IND; 4 Pediatrics, University of South Alabama Children’s and Women’s Hospital, Mobile, USA; 5 Internal Medicine, Sidra Hospital, Doha, QAT; 6 Cardiothoracic Surgery, University of Alabama at Birmingham, Birmingham, USA; 7 Family Medicine, University of North Dakota School of Medicine and Health Sciences, Grand Forks, USA; 8 Research and Development, Shing Huei Group, Taipei, TWN; 9 Cardiology, Pakistan Navy Station (PNS) Shifa, Karachi, PAK

**Keywords:** bronchiolitis, cpap, high-flow nasal cannula, pediatric respiratory failure, respiratory support

## Abstract

This systematic review and meta-analysis compared the efficacy of continuous positive airway pressure (CPAP) and high-flow nasal cannula (HFNC) therapy in children with acute bronchiolitis. A comprehensive literature search across multiple electronic databases identified six randomized controlled trials for inclusion. The primary outcomes assessed were treatment failure, the need for invasive mechanical ventilation, and length of hospital stay. Pooled analysis revealed no significant difference between CPAP and HFNC in the risk of requiring invasive mechanical ventilation (RR 0.94, 95% CI: 0.60-1.46) with minimal heterogeneity across studies. Treatment failure was higher in the HFNC group than in CPAP, but this difference was not statistically significant (RR 1.20, 95% CI: 0.63-2.27), though heterogeneity was substantial (I²=70%). Sensitivity analysis after removing one study showed a significantly higher risk of treatment failure with HFNC (RR 1.67, 95% CI: 1.07-2.61) with reduced heterogeneity. Length of hospital stay was comparable between both interventions (MD 0.57, 95% CI: -0.16-1.31). Both respiratory support strategies effectively reduce respiratory effort in moderate to severe bronchiolitis through different mechanisms, such as CPAP, which provides consistent positive end-expiratory pressure, and HFNC through enhanced minute ventilation and nasopharyngeal dead space reduction. Despite some limitations, including small sample sizes and inability to conduct subgroup analyses due to lack of individual patient data, this meta-analysis suggests HFNC may serve as a viable alternative to CPAP, particularly in resource-limited settings, showing comparable outcomes for critical endpoints while potentially offering practical advantages in administration and patient comfort.

## Introduction and background

The respiratory syncytial virus (RSV) is the main cause of acute bronchiolitis, the most prevalent lower respiratory tract illness in newborns and young children [1]. It is a leading cause of hospital admissions in children under two years of age, often presenting with symptoms of cough, wheezing, tachypnea, and respiratory distress [2]. While most cases are mild and self-limiting, a subset of infants develops severe respiratory distress requiring respiratory support [3]. The optimal non-invasive respiratory support strategy for acute bronchiolitis remains a subject of ongoing debate, particularly between high-flow nasal cannula (HFNC) therapy and continuous positive airway pressure (CPAP) [4]. 

HFNC has gained widespread use in recent years due to its ease of administration, patient comfort, and ability to deliver heated, humidified oxygen at high flow rates, generating a degree of positive end-expiratory pressure (PEEP) [5,6]. It has been shown to improve oxygenation and reduce the work of breathing by providing flow-dependent airway distension and dead space washout [7]. Despite these advantages, concerns remain regarding its effectiveness in preventing escalation to more invasive respiratory support compared to CPAP.

CPAP, on the other hand, has been used for decades in pediatric respiratory failure and is recognized for its ability to provide consistent PEEP, reduce atelectasis, and improve functional residual capacity [8]. By maintaining airway patency and reducing inspiratory effort, CPAP may offer superior respiratory support in children with moderate to severe bronchiolitis [9]. However, CPAP is often associated with greater patient discomfort, the need for sedation in some cases, and a higher level of nursing and medical supervision compared to HFNC [10]. 

Recent studies have attempted to compare the efficacy and safety of CPAP and HFNC in managing acute bronchiolitis, but results have been inconsistent. While some studies suggest that HFNC is non-inferior to CPAP in preventing treatment failure and reducing intensive care unit (ICU) admissions, others report superior outcomes with CPAP in reducing respiratory distress and avoiding intubation [11-14]. Given the clinical and practical implications of selecting the optimal respiratory support strategy in children with acute bronchiolitis, this systematic review and meta-analysis aim to synthesize the available evidence comparing CPAP and HFNC in terms of efficacy outcomes. By analyzing treatment failure rates, the need for intubation, and the length of hospital stay, our study will provide valuable insights to guide clinical decision-making and optimize respiratory management strategies for children with acute bronchiolitis.

## Review

Methodology 

Literature Search and Search Strategy 

A comprehensive literature search was conducted to identify relevant studies comparing CPAP and HFNC in children with acute bronchiolitis. The search was performed across multiple electronic databases, including MedLine (PubMed), Embase, Web of Science, Cochrane Library, and Scopus. The search strategy employed a systematic approach to identify relevant studies comparing CPAP and HFNC in pediatric bronchiolitis. Medical Subject Headings (MeSH) terms, including "Bronchiolitis," "Bronchiolitis, Viral," "Respiratory Syncytial Virus Infections," "Continuous Positive Airway Pressure," "Positive-Pressure Respiration," "Cannula," and "Oxygen Inhalation Therapy" were combined with free-text keywords such as "bronchiolitis," "respiratory syncytial virus," "RSV," "CPAP," "continuous positive airway pressure," "high flow nasal cannula," "HFNC," "high-flow oxygen therapy," and "nasal oxygen." Population terms like "infant," "child," "pediatric," and "paediatric" were incorporated to focus on the relevant age group. Boolean operators (AND, OR) connected these terms to capture all potential studies examining these interventions. The search was further refined by applying filters for randomized controlled trials and using terms like "randomized," "randomised," "controlled trial," and "comparative effectiveness research." No language restrictions were applied, and the search was limited to studies published up to 25 January 2025. Reference lists of included articles and relevant review papers were also manually screened to identify additional eligible studies. The search was performed by two authors independently. Disagreement that occurred during the process of the literature search was resolved through consensus, and the principal investigator was involved in the discussion if required.

Study Selection 

Studies were included if they met the following criteria: infants and children diagnosed with acute bronchiolitis; studies comparing CPAP and HFNC; reporting of at least one of the following clinical outcomes-treatment failure, need for intubation, length of hospital stay, or adverse events; and study design limited to randomized controlled trials (RCTs). Studies involving patients with significant comorbidities unrelated to bronchiolitis were excluded. Case reports, observational studies, conference abstracts without sufficient data, and editorials were also excluded from this study. Following a screening of all identified studies' titles and abstracts by two independent reviewers, full-text papers of possibly suitable studies were obtained for additional evaluation. Predetermined eligibility criteria were used to decide the final inclusion. A third reviewer was consulted or discussed with in order to settle any disputes between the reviewers. The Preferred Reporting Items for Systematic Reviews and Meta-Analyses (PRISMA) flow diagram was used to record the study selection procedure [15].

Data Extraction and Quality Assessment 

Data from the included studies were extracted using a pre-designed data extraction form on Microsoft Excel (Redmond, USA). Extracted information included study characteristics (author, year of publication, country, study design, and sample size) and reported outcomes (treatment failure, intubation rate, length of hospital stays, and adverse events). The quality of the included studies was assessed using the Cochrane Risk of Bias (RoB) tool [16]. Two independent reviewers conducted the data extraction and performed the quality assessment, and any discrepancies were resolved by consensus.

For evidence quality assessment, we implemented the Grading of Recommendations, Assessment, Development, and Evaluations (GRADE) methodology. GRADEPro GDT was used to perform the GRADE assessment (McMaster University and Evidence Prime, 2025) [17].

Data Analysis 

RevMan Version 5.4.1 was used for the data analysis. We computed the risk ratio (RR) with a 95% confidence interval (CI) for categorical outcomes, such as the risk of invasive mechanical ventilation and treatment failure. We presented the mean difference (MD) with a 95% confidence interval (CI) for continuous outcomes, including the duration of hospitalization. P-values below 0.05 were regarded as statistically significant. Cochran's Q test and the I² statistic were used to evaluate heterogeneity. Low heterogeneity was indicated by an I² value of 25% or less, moderate heterogeneity by values between 25% and 50%, and high heterogeneity by values more than 50%. We performed a sensitivity analysis to assess the influence of specific studies on the overall findings to investigate potential causes of heterogeneity. 

Results 

**Figure 1:**
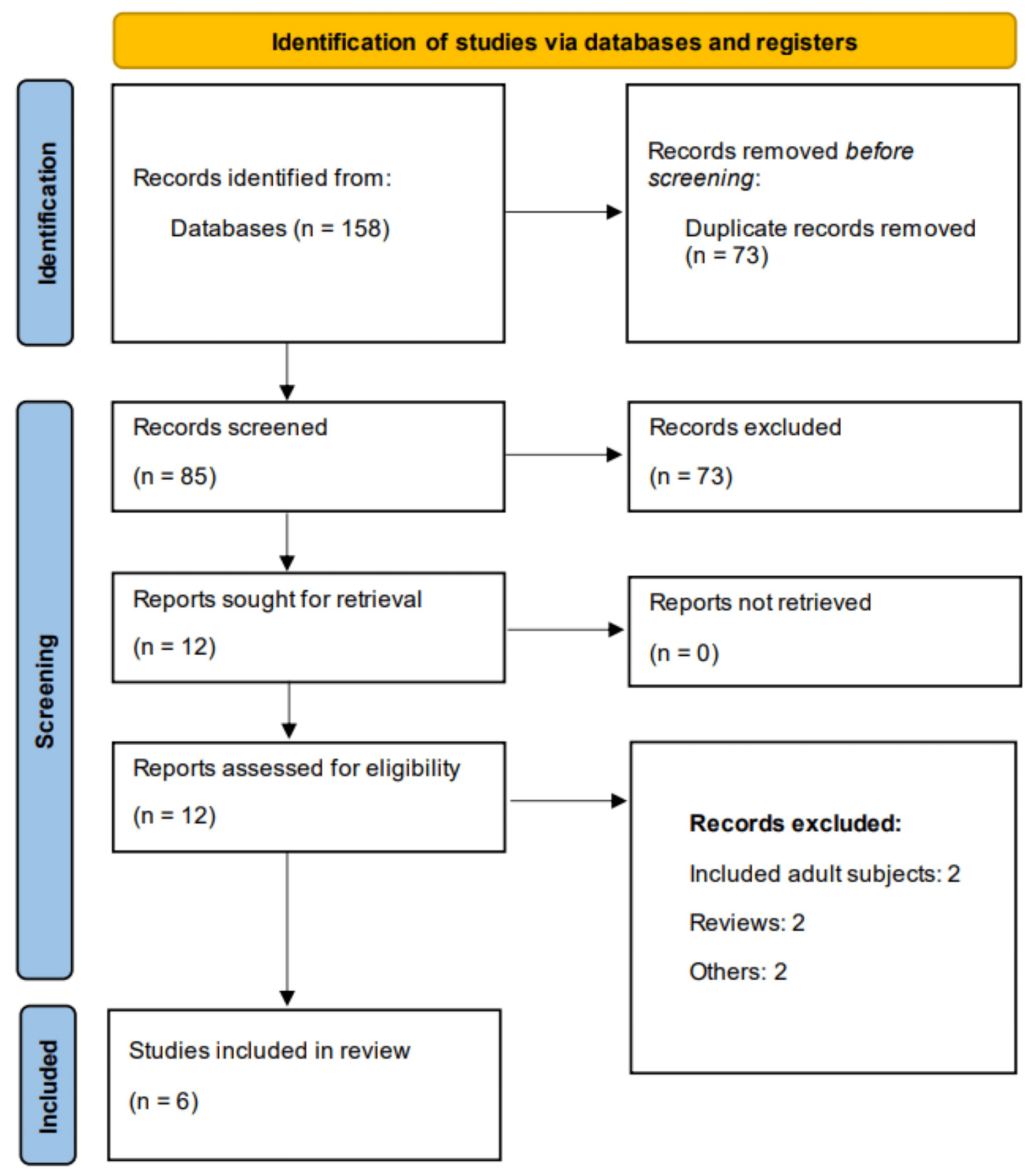
PRISMA flowchart (study selection process)

**Figure 2:**
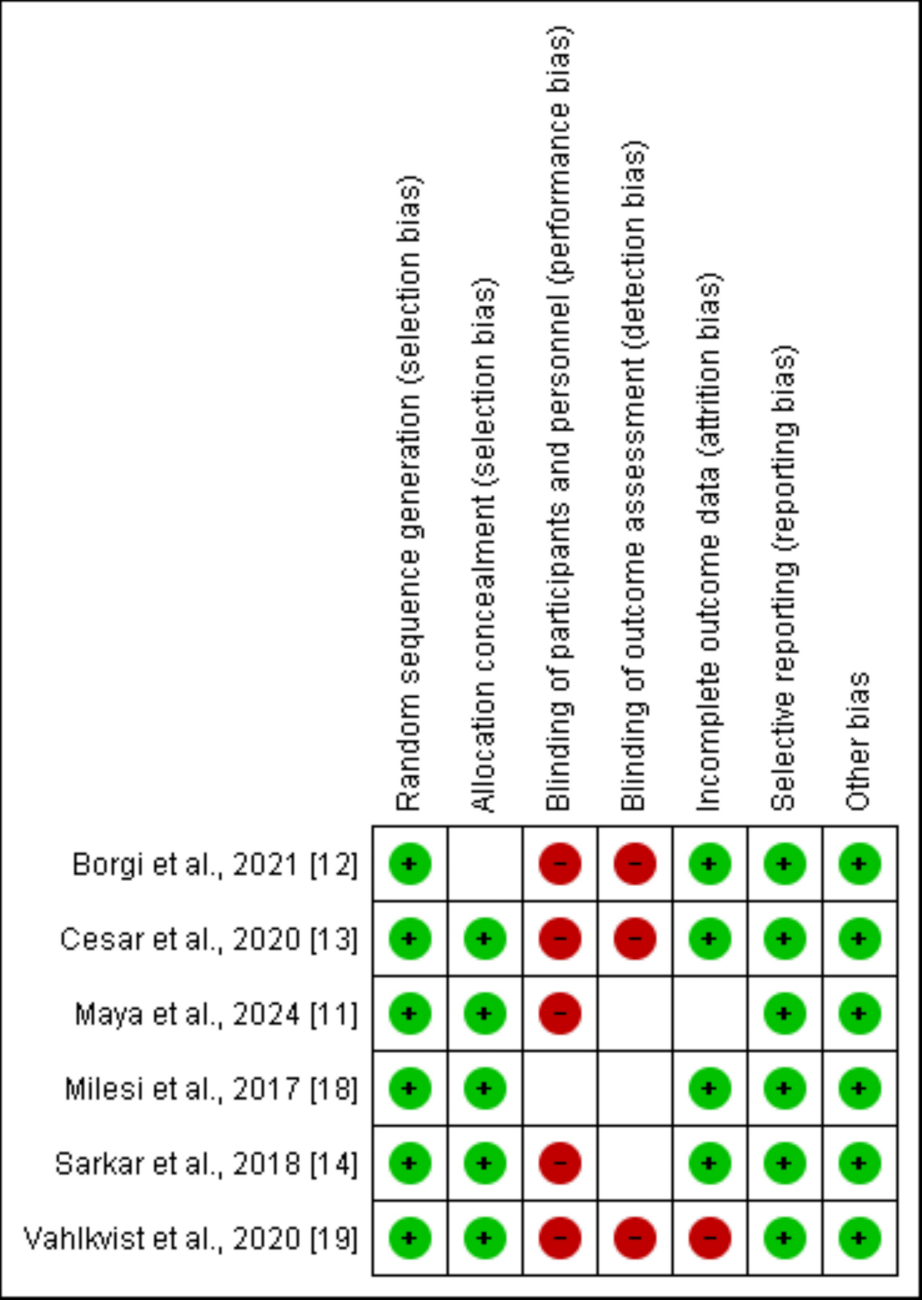
Quality assessment of included studies

**Figure 3:**
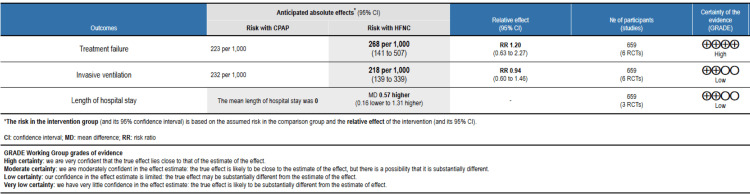
GRADE summary of findings table with all the outcomes

Through online database searching, we found 158 records. After removing duplicates, the initial screening of 85 records was done by two authors. Detailed assessment was done after obtaining the full text of 12 articles. Finally, six articles were included in this meta-analysis. Figure 1 shows the study selection process. Table 1 presents the characteristics of the included studies. Figure 2 presents a quality assessment of all included studies. Figure 3 presents a GRADE assessment of all included studies. Our evidence synthesis indicates that while there is high certainty that treatment failure rates between HFNC and CPAP may differ (RR 1.20, 95% CI 0.63-2.27), the confidence interval spans both benefit and harm. For invasive mechanical ventilation, low-certainty evidence suggests comparable effectiveness (RR 0.94, 95% CI 0.60-1.46), while high-certainty evidence indicates a non-significant trend toward slightly longer hospital stays with HFNC. These findings highlight the importance of considering both the point estimates and their uncertainty when selecting respiratory support modalities.

**Table 1:** Characteristics of included studies CPAP: Continuous Positive Airway Pressure HFNC: High-Flow Nasal Cannula

Author	Year	Setting	Region	Groups	Sample Size	Age (in Months)
Borgi et al. [12]	2021	Single center	Tunisia	HFNC	130	1.78
				CPAP	125	1.62
Cesar et al. [13]	2020	Single center	Brazil	HFNC	35	3.37
				CPAP	28	2.43
Maya et al. [11]	2024	Single center	India	HFNC	59	7
				CPAP	59	4
Milesi et al. [18]	2017	Multi-center	France	HFNC	71	1.4
				CPAP	71	1.27
Sarkar et al. [14]	2018	Single center	India	HFNC	15	4.06
				CPAP	16	2.81
Vahlkvist et al. [19]	2020	Single center	Denmark	HFNC	22	2.1
				CPAP	28	2.8

Meta-analysis of outcomes 

*Invasive Mechanical Ventilation* 

**Figure 4:**
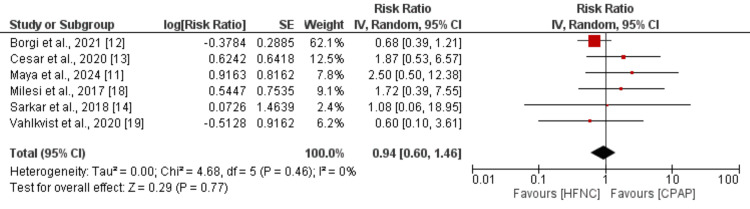
Comparing risk of invasive ventilation between two groups References [11-14,18-19]

Six studies were included in the pooled analysis comparing the risk of invasive mechanical ventilation between children in the CPAP and HFNC groups, and the results of the pooled analysis are shown in Figure 4. Pooled analysis indicated that the risk of mechanical ventilation was not significantly different between the two groups (0.94, 95% CI: 0.60 to 1.46). No significant heterogeneity was reported among the study results (I-square: 0%, p-value: 0.46). 

Treatment Failure 

**Figure 5:**
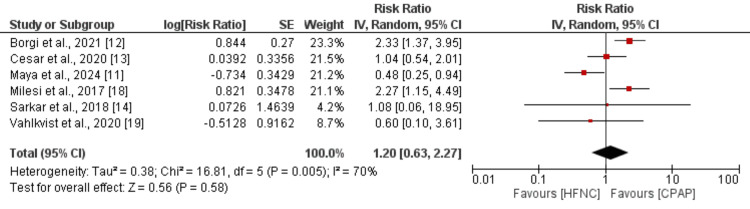
Comparison of treatment failure between two groups References [11-14,18-19]

Six studies were included in the pooled analysis comparing the risk of treatment failure between children in the CPAP and HFNC groups, and the results of the pooled analysis are shown in Figure 5. Pooled analysis showed that the risk of treatment failure was higher in the HFNC group compared to the CPAP group, but the difference was statistically insignificant (1.20, 95% CI: 0.63 to 2.27). Significant heterogeneity was reported among the study results (I-square: 70%, p-value: 0.005).

Length of Hospital Stay in Days 

**Figure 6:**
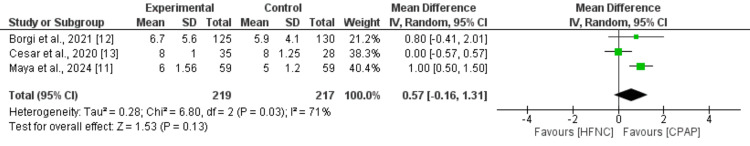
Comparison of length of hospital stay between two groups References [11-13]

Three studies were included in the pooled analysis comparing the length of hospital stay between children in the CPAP and HFNC groups, and the results of the pooled analysis are shown in Figure 6. As shown in Figure 4, the length of hospital stay was not different between the two groups (MD: 0.57, 95% CI: -0.16 to 1.31). High heterogeneity was reported among the study results (I-square: 71%, p-value: 0.03).

Sensitivity analysis

Two of the three outcomes assessed in this study, treatment failure and length of hospital stay, showed significant heterogeneity. We performed sensitivity analysis by removing the study conducted by Maya et al. [11]. Although the risk of treatment failure remained higher in children in the HFNC group, the difference became statistically significant (RR: 1.67, 95% CI: 1.07 to 2.61). Heterogeneity decreased substantially from 70% to 28%. Similarly, when computing the effect estimate of the length of hospital stay without the Maya et al. study [11], although hospital stays remained longer in the HFNC group (MD: 0.22, 95% CI: -0.48 to 0.91), heterogeneity decreased markedly from 71% to 27%.

The Maya et al. study [11] included a notably broader age range (1-23 months, median age seven months in the HFNC group) compared to other studies that primarily enrolled younger infants (median ages 1.4-4.1 months). This age difference is clinically significant, as bronchiolitis pathophysiology and airway mechanics vary substantially between young infants and older children. Younger infants typically have smaller airways with higher resistance and greater susceptibility to mucosal edema, potentially responding differently to pressure-based therapies like b-CPAP versus flow-based therapies like HFNC. This age heterogeneity likely explains Maya's divergent findings favoring HFNC, as older infants might benefit more from HFNC's flow-dependent mechanisms than from the constant pressure provided by CPAP.

Discussion 

This meta-analysis included six RCTs comparing outcomes between CPAP and HFNC, assessing the risk of therapeutic failure and the need for invasive mechanical ventilation. The findings indicated no significant difference between the two groups in terms of treatment failure rates. However, considerable heterogeneity was observed across studies, requiring careful assessment of both clinical and statistical factors influencing these outcomes.

Sources of Heterogeneity

Our comprehensive assessment of heterogeneity revealed multiple contributing factors. Statistically, the I² values were substantial for treatment failure (70%) and length of hospital stay (71%), suggesting important variability in effect estimates across studies. Through systematic evaluation of forest plots and influence analyses, we identified that the Maya et al. study [11] significantly contributed to this heterogeneity. When excluded in sensitivity analyses, heterogeneity decreased markedly (I² reduced to 28% for treatment failure and 27% for length of stay).

Clinically, several factors likely contributed to between-study variability. First, substantial differences in patient populations existed, with Maya et al. [11] including older infants (median age seven months) compared to other studies (median ages 1.4-4.1 months). Second, the studies employed different CPAP delivery methods: Maya utilized bubble CPAP while others used ventilator-driven CPAP systems. Third, treatment failure definitions varied considerably across studies, from objective criteria like specific changes in respiratory rate to more subjective clinical assessments. Fourth, healthcare settings differed substantially, with studies conducted in varied resource contexts that may have influenced overall care approaches [20].

Contextualizing Findings

These results contrast with previous studies, including a systematic review and meta-analysis of five RCTs conducted up to June 2022, which focused on children under two years with moderate to severe bronchiolitis and reported different conclusions [21]. Our review incorporated a more recent RCT comparing HFNC and CPAP, which was the only study to report a significantly lower treatment failure rate in the HFNC group compared to CPAP. Additionally, we found no significant difference in the length of hospital stay between the two interventions. However, Maya et al. [11] reported a longer hospital stay for patients in the HFNC group compared to CPAP. 

HFNC has been compared in numerous trials with other respiratory support techniques, such as B-CPAP, CPAP, nasal positive pressure ventilation, and normal oxygen therapy. These studies evaluated the severity of respiratory diseases in various pediatric care settings and reported a variety of results [22-26]. For instance, an RCT conducted between 2012 and 2015 evaluated high-flow warm humidified oxygen against standard nasal cannula oxygen in children under 24 months with moderate bronchiolitis [22]. The findings indicated that treatment failure was less common in the HFNC group compared to standard oxygen therapy. However, HFNC did not lead to a shorter duration of oxygen therapy in the emergency department.

Challenges in Outcome Assessment

Interpreting treatment failure requires careful consideration, as its definition varies across studies. In the Cesar et al. (2020) study [13], treatment failure was defined as the need for escalation to bilevel noninvasive ventilation or endotracheal intubation. Moreover, determining treatment failure often involves a degree of subjectivity, as it is not solely based on objective ventilatory parameters. This heterogeneity in outcome definitions represents a significant methodological challenge when comparing efficacy across studies.

A more reliable way to validate outcomes like treatment failure may be to examine their correlation with objective measures such as intubation rates or mortality. In this analysis, CPAP did not demonstrate a lower risk of mortality or mechanical ventilation. The inherent subjectivity in defining treatment failure across studies may have introduced bias, making it a less dependable measure for evaluating the effectiveness of these respiratory support strategies.

Physiological Mechanisms

Given the pathophysiology of respiratory failure in bronchiolitis, both CPAP and HFNC serve as effective interventions to reduce respiratory effort in patients with moderate to severe lung disease. This, in turn, helps lower the need for invasive ventilatory support and its associated complications [26,27]. The proposed mechanism of action of CPAP is based on the concept that positive end-expiratory pressure (PEEP) enhances residual functional capacity and lung volume, preventing alveolar collapse. Additionally, it widens the airways, thereby reducing lower airway resistance and preventing obstructive apnea, which is particularly beneficial for patients with bronchiolitis [26]. For HFNC, delivering heated and humidified gas at a high flow enhances minute ventilation and reduces dead space in the nasopharynx, thereby decreasing respiratory effort. This process also facilitates secretion clearance while preventing bronchial obstruction and inflammation caused by exposure to dry, cold air. Additionally, HFNC is believed to generate a certain level of PEEP, although it is less than that provided by CPAP and cannot be precisely measured [28].

Clinical Implications

Based on the findings of our meta-analysis and careful consideration of between-study heterogeneity, there were no significant differences in mortality or the need for mechanical ventilation between HFNC and CPAP in children with severe bronchiolitis. HFNC was associated with a lower risk of adverse events, which may make it a potentially favorable option in certain clinical contexts [29]. The substantial heterogeneity observed suggests that patient-specific factors, healthcare setting characteristics, and implementation approaches may significantly influence comparative effectiveness.

While both interventions have been effective in reducing intubation rates in previous studies, our analysis reinforces the need for further high-quality clinical trials with standardized outcome definitions to confirm these outcomes, particularly in diverse clinical settings, including resource-limited environments. Future research should emphasize age-stratified analyses and clearly defined, objective outcome measures to better clarify which patient populations might derive greater benefit from each intervention.

Study limitations

This meta-analysis has several limitations that should be acknowledged. First, the overall sample size across the included studies is relatively small, which may limit the statistical power and generalizability of the findings. Additionally, the number of studies available for inclusion is limited, restricting the robustness of the conclusions drawn. Second, the lack of access to individual patient-level data prevented us from conducting subgroup analyses. As a result, we were unable to assess whether the efficacy of different interventions varied across specific patient subgroups, such as those with differing disease severity, age groups, or comorbidities. Subgroup analyses could have provided valuable insights into which populations may benefit more from HFNC or CPAP, further refining clinical decision-making. Third, many of the included studies had relatively short follow-up durations, limiting our ability to evaluate long-term outcomes. There was also considerable variability in how key outcomes, such as treatment failure, were defined across studies, which could contribute to heterogeneity and affect the comparability of results. Finally, none of the studies included a cost-effectiveness assessment, which is an important consideration for informing healthcare policy and clinical practice. Future research with larger, well-powered studies, standardized outcome definitions, longer follow-up periods, and access to granular patient-level data is essential to address these gaps and enhance the precision and applicability of treatment recommendations.

Our findings warrant careful consideration of imprecision and evidence fragility. The wide confidence intervals for treatment failure (RR 1.20, 95% CI 0.63-2.27) and mechanical ventilation (RR 0.94, 95% CI 0.60-1.46) span both potential benefit and harm, reflecting the modest cumulative sample size (659 participants) and outcome heterogeneity. With only 88 total ventilation events across studies, even small outcome shifts could alter conclusions. The optimal information size for detecting meaningful differences would require approximately 800-1,000 patients-substantially more than available. These limitations underscore the need for larger trials with standardized outcomes before drawing definitive conclusions about HFNC versus CPAP efficacy.

## Conclusions

This meta-analysis demonstrates that CPAP and HFNC show comparable efficacy in preventing invasive mechanical ventilation in children with acute bronchiolitis, with no significant difference in length of hospital stay. While our initial analysis showed no significant difference in treatment failure rates, sensitivity analysis revealed potentially higher treatment failure with HFNC. Both modalities effectively reduce respiratory effort through different physiological mechanisms. HFNC may serve as a viable alternative to CPAP, particularly in resource-limited settings, offering practical advantages in administration and patient comfort despite potentially higher treatment failure rates. Study limitations include small sample sizes and the inability to conduct subgroup analyses. Future research with larger cohorts and access to individual patient data is essential to refine treatment recommendations based on specific patient characteristics and disease severity.
